# Intense Caloric Restriction from Birth Protects the Heart Against Ischemia/Reperfusion Injury and Reduces Reactive Oxygen Species in Ovariectomized Rats

**DOI:** 10.3390/antiox14020169

**Published:** 2025-01-31

**Authors:** Vinícius Lopes Cantuária, Cíntia Maria Rodrigues, Isabella Rocha Dias, Vinícius de Oliveira Ottone, Bruna Oliveira Costa, Lourdes Fernanda Godinho, Gabriela Silva, Marco Antônio Alves Schetino, Etel Rocha-Vieira, Marco Fabrício Dias-Peixoto, Kinulpe Honorato-Sampaio

**Affiliations:** 1 Faculdade de Medicina, Universidade Federal dos Vales do Jequitinhonha e Mucuri, Diamantina 39100-000, MG, Brazil; vinicius.cantuaria@ufvjm.edu.br (V.L.C.); vinicius.ottone@ufvjm.edu.br (V.d.O.O.); marco.schetino@ufvjm.edu.br (M.A.A.S.); etel.vieira@ufvjm.edu.br (E.R.-V.); 2 Programa de Pós-Graduação em Ciências da Saúde, Universidade Federal dos Vales do Jequitinhonha e Mucuri, Diamantina 39100-000, MG, Brazil; bruna.costa@ufvjm.edu.br (B.O.C.); lourdes.godinho@ufvjm.edu.br (L.F.G.); gabriela.silva@ufvjm.edu.br (G.S.); marcofabri@ufvjm.edu.br (M.F.D.-P.); 3 Faculdade de Ciências Biológicas e da Saúde, Universidade Federal dos Vales do Jequitinhonha e Mucuri, Diamantina 39100-000, MG, Brazil; cintia.rodrigues@ufvjm.edu.br (C.M.R.); isabella.dias@ufvjm.edu.br (I.R.D.)

**Keywords:** menopause, diet, mitochondria, oxidative stress

## Abstract

This study investigates the cardioprotective effects of intense caloric restriction (ICR) from birth in ovariectomized rats, a model of estrogen deficiency mimicking menopause. Our findings demonstrate that ICR significantly improved both basal and post-ischemic cardiac function, even in the absence of estrogens. The restricted animals exhibited enhanced cardiac contractility and relaxation, particularly after ischemia/reperfusion (I/R) injury, with superior functional recovery compared to control groups. Notably, ICR reduced key cardiometabolic risk factors, including blood pressure, heart rate, and adiposity, while improving glucose tolerance and insulin sensitivity. Additionally, while mitochondrial biogenesis remained unaffected, ICR preserved mitochondrial integrity by reducing the number of damaged mitochondria. This was linked to a reduction in oxidative stress, as evidenced by lower reactive oxygen species (ROS) production in the hearts of restricted animals. These results suggest that ICR offers a protective effect against cardiovascular dysfunction induced by estrogen depletion, potentially through enhanced antioxidant defenses and mitochondrial protection.

## 1. Introduction

Menopause is a significant event in a woman’s life cycle, marking the permanent cessation of menstruation. It typically occurs between the ages of 40 and 55 as a result of the physiological degeneration of ovarian follicles [1,2], accompanied by a decline in sex hormone levels, particularly estrogens [3]. This reduction in ovarian hormone levels contributes to cardiometabolic alterations [4], increasing the risk of cardiovascular diseases [5,6].

Studies have shown that estrogens, particularly 17β-estradiol (E2), play a protective role in the cardiovascular system [7,8], acting as vasoactive agents with antiarrhythmic, vasodilatory, and notably antioxidant properties [9]. The ovariectomized rat model, widely used to replicate the estrogen reduction observed in human menopause [10], has demonstrated that E2 supplementation reduces ischemia/reperfusion (I/R) cardiac injury [11,12], lowers blood pressure [13], improves vascular function through estrogen receptors in endothelial, smooth muscle, and vascular cells [14], and enhances redox status by reducing reactive oxygen species (ROS) levels [15].

Given these findings, the cardioprotective effects of estrogens are evident [16]. The absence of estrogens, as seen in menopause and postmenopause, is a contributing factor to the development of cardiovascular diseases [17,18,19]. While some studies have highlighted the benefits of hormone replacement therapy (HRT) on cardiovascular function, the results remain controversial, and HRT is not recommended for all women [20].

Caloric restriction (CR) is an effective non-pharmacological intervention that reduces cardiometabolic risk factors and prevents cardiovascular diseases [21,22]. Caloric restriction has also been shown to improve health and extend the lifespan [23]. Some authors suggest that the effects of CR may be either beneficial or not, depending on the timing and intensity of its initiation [24,25]. According to Han and Ren (2010), the benefits of CR are more pronounced the earlier it is implemented [21]. A previous study with male Wistar rats subjected to 50% CR from birth reported positive adaptations in cardiac function, morphology, and signaling pathways when compared to the ad libitum group [26]. Furthermore, this intervention had positive effects on cardiac function following I/R injury [27]. In elderly animals, intense caloric restriction (ICR) reduced the expression of inflammatory markers and cardiovascular risk factors (e.g., blood glucose, triglycerides), reduced cardiac hypertrophy and arrhythmia associated with aging, and enhanced antioxidant defense through an increased superoxide dismutase (SOD) activity [28].

Considering the cardioprotective effects of estradiol in females, we hypothesized that intense caloric restriction from birth could also protect the heart in the absence of estrogens. Thus, we initially demonstrated that ICR from birth improved cardiac function in ovariectomized rats [29]. Building on our previous findings, the present study investigated whether this same intervention could protect hearts from ischemia/reperfusion injury, while also evaluating cardiovascular risk factors, mitochondrial content, and reactive oxygen species production.

## 2. Materials and Methods

### 2.1. Animals and Caloric Restriction Protocol

The experimental protocol was approved on 19 February 2020 by the Animal Ethics Committee (CEUA) of the Universidade Federal dos Vales do Jequitinhonha e Mucuri (UFVJM), Diamantina, MG, Brazil (protocol 058/2019), and was performed in accordance with the Guide for the Care and Use of Laboratory Animals published by the U.S. National Institutes of Health [30].

The animals were obtained from the animal facility of the Federal University of Viçosa (Brazil). The caloric restriction protocol followed previous studies [26,27,28,29]. In brief, healthy pregnant Wistar rats (*n* = 12), aged 3 months, were housed in individual cages under controlled light and temperature conditions (22 ± 2 °C; inverted 12 h light–dark cycle), with free access to water and a commercial diet containing 22.5% protein, 4% fat, 54% carbohydrates, 8% fiber, and 12.5% moisture (Nuvilab Nutrients LTDA, Colombo, PR, Brazil). Immediately after the birth of the offspring, the dams were randomly divided into two groups: half (*n* = 6) were assigned to the control group (C) and the other half (*n* = 6) were assigned to the restriction group (R). The dams in the control group were given ad libitum access to food, while the dams in the restriction group were fed daily with 50% of the amount consumed by the control group. Each litter consisted of the mother and 8 pups. After weaning (3 weeks after birth), the female offspring were given the same treatment as their mothers. At 12 weeks of age, half of the rats in each group (C and R) underwent bilateral ovariectomy (OVX), resulting in a total of 4 experimental groups: C-Sham, C-OVX, R-Sham, and R-OVX. The animals were maintained on their respective dietary treatments for an additional 4 weeks, after which metabolic function was assessed using oral glucose tolerance (OGTT) and intraperitoneal insulin tolerance (IITT) tests. Cardiovascular function was evaluated by plethysmography and the Langendorff isolated heart method. All females entered puberty and sexual maturation prior to ovariectomy. Body weight was recorded weekly from birth, puberty was confirmed by vaginal opening and estrous cycle monitoring through daily vaginal smears, as reported in previous studies [29,31].

### 2.2. Ovariectomy

The rats were weighed and anesthetized via intraperitoneal injection of ketamine (116 mg/kg) and xylazine (6 mg/kg). Ovariectomy was conducted through bilateral incisions in the skin and muscle layers on the flanks, parallel and lateral to the spine from the mid-thoracic curvature to end of curvature, until the abdominal cavity. The fat pad surrounding the ovary was exteriorized using forceps, and mosquito hemostats were applied to crush the cranial portion of the uterine horn distal to the ovary. The ovary was then removed, and the hemostats were kept clamped for 30 s to minimize hemorrhaging. Subsequently, the uterine horn was returned to the abdominal cavity, and the skin incision was sutured. In the sham group, the ovaries were left intact. Postoperative care included administration of Pentabiotic (24,000 IU/kg, intramuscular; Fort Dodge) and Flunixin Meglumine (2.5 mg/kg, subcutaneous). The animals were monitored for 7 days after surgery to assess their well-being. The success of the ovariectomy was verified by a decrease in uterine mass and plasma E2 levels [29].

### 2.3. Oral Glucose Tolerance Test (OGTT)

The animals were fasted for 8 h, followed by gavage with an anhydrous dextrose solution (2 g/kg body weight, 50% solution). Circulating glucose concentrations were determined by making a small incision in the tail for blood collection. Glucose levels were monitored before dextrose administration (time 0) and after 15, 30, 60, and 120 min. Circulating glucose values were determined using an ACCU-CHEK glucometer (Advantage Glucose Analyzer, Roche Diagnostics Corporation, Indianapolis, IN, USA). From the data, glucose curves were constructed over time, and the area under the curve (AUC) was calculated.

### 2.4. Intraperitoneal Insulin Tolerance Test (IITT)

Forty-eight hours after the oral glucose tolerance test, the animals underwent a twelve h fast, followed by an intraperitoneal injection of insulin (Humulin R—recombinant DNA-derived, 100 U/mL, dose: 1 U/kg body weight). Blood glucose concentrations were determined following the same protocol as the oral glucose tolerance test.

### 2.5. Plethysmography

The animals were acclimated to tail-cuff plethysmography over a period of 5 days using an MLT1020PPG IR Plethysmograph (ADInstruments, Bella Vista, NSW, Australia). Systolic blood pressure (SBP) and heart rate (HR) measurements were recorded, and the double product (DP) index was calculated as the product of SBP and HR (SBP × HR).

### 2.6. Euthanasia and Sample Collection

Forty-eight hours after the insulin tolerance test, the animals were fasted for eight h and euthanized by decapitation. Blood samples were collected for biochemical (total cholesterol, HDL, and triglycerides) and hormonal analyses. The heart was rapidly excised and connected to the Langendorff perfusion system for cardiac function assessment. Additionally, the uterus, liver, and retroperitoneal and parametrial adipose tissues were dissected and weighed.

### 2.7. Hormonal and Biochemical Analyses

Blood was collected in tubes without anticoagulants. After collection, the blood was centrifuged at 1800 rpm to obtain serum samples, which were used to determine the serum concentrations of total cholesterol and its fractions, triglycerides, and estradiol. Biochemical analyses were conducted using kits from Labtest Diagnóstica LTDA (Lagoa Santa, MG, Brazil), and E2 levels were measured by ELISA with a commercial kit from Neogen Corporation (Lexington, KY, USA), following the manufacturer’s recommended procedures. The anti-E2 antibody cross-reacts 1% with testosterone, 0.4% with estriol, 0.1% with estrone, 0.03% with dehydroepiandrosterone, and <0.02% with other steroid hormones.

### 2.8. Ex Vivo Cardiac Function Analysis

Cardiac function was assessed using the Langendorff perfusion system (ML785B2, ADInstruments). Rats were rapidly decapitated, the chest was opened, and the heart was excised and retrogradely perfused through the Langendorff apparatus with Krebs–Ringer solution (in mmol·L^−1^: NaCl, 118.40; KCl, 4.70; KH_2_PO_4_, 1.17; MgSO_4_, 1.17; CaCl_2_, 2.50; glucose, 11.65; NaHCO_3_, 26.30) at 37 ± 1 °C, under constant pressure (65 mmHg) and oxygenation (5% CO_2_ and 95% O_2_). Contractility (+dP/dt), relaxation (−dP/dt), and heart rate (HR) were calculated using AcqKnowledge software [27] (Version 8.1.30, TSD 104A, Biopac Systems Inc., Santa Barbara, CA, USA). After stabilization in the perfusion system, basal cardiac function was assessed for 15 min. The hearts were then subjected to 10 min of global ischemia. Following ischemia, hearts were reperfused for 15 min for post-ischemic cardiac function evaluation. All ±dP/dt measurements were normalized by heart weight [19,32,33]. After the experiment, left ventricles were excised, and samples were fixed overnight in a modified Karnovsky fixative (2.5% glutaraldehyde and 2% paraformaldehyde in 0.1 M cacodylate buffer, pH 7.4) at 4 °C. The remaining ventricular tissue was frozen in liquid nitrogen and stored at −80 °C (Indrel^®^ IULT 486D, Londrina, PR, Brazil) for molecular analyses.

### 2.9. Histological Analysis

Samples of retroperitoneal adipose tissue from 5 animals per group were fixed in 4% paraformaldehyde and embedded in paraffin (Histotec Pastilles—Merck, Darmstadt, Germany) at 60–65 °C (Lucadema^®^ Oven). Sections of 5 μm were cut using a microtome (YD-355AT^®^) and stained with hematoxylin and eosin. Images at 20× magnification were acquired using a Nikon Eclipse 200 microscope (Tokyo, Japan). Adipocyte areas were quantified using ImageJ software (Version 1.53, National Institutes of Health, Bethesda, MD, USA). One hundred cells per animal were evaluated [18].

### 2.10. Transmission Electron Microscopy

Samples of the left ventricle fixed in the Karnovsky fixative were post-fixed with 1% osmium tetroxide, reduced with potassium ferrocyanide, and subjected to in-block contrast with uranyl acetate. The samples were embedded in EPON 812, sectioned to 50 nm thickness, and contrasted with lead nitrate. Morphometric analyses were performed as described previously [17,34]. In brief, the mitochondrial volume density was analyzed by point counting, and the mitochondrial area was measured using ImageJ software (Version 1.53, National Institutes of Health). We analyzed 72 cells/group.

### 2.11. Real-Time PCR

Total RNA was extracted from frozen cardiac tissue using TRI Reagent (Sigma^®^). Briefly, the tissue was homogenized in TRI Reagent, followed by phase separation with chloroform, and RNA was precipitated with isopropanol. The RNA pellet was washed with 75% ethanol, dried, and dissolved in RNase-free water. For reverse transcription, we incubated a reaction mix containing 9 µL sample, 4 µL Script RT buffer, 4 µL H_2_O RNAse Free, 1 µL dNTP Mix, 1 µL DTT stock, 0.5 µL Primer Randon, 0.5 µL RNAse Inhibitor, and 0.5 µL Script Reverse Transcriptase (Cellco^®^) in a thermal cycler (StepOne PCR system, Applied Biosystems, Foster City, CA, USA) under the following conditions: 10 min at 42 °C, 40 min at 55 °C, and 10 min at 70 °C.

Real-time PCR was carried out to amplify cDNA using specific primers for the gene of *Pgc-1α* and the housekeeping gene *Gapdh* as a normalizer: (1) *Pgc-1α* sense 5′→3′ ATGTGTCGCCTTCTTGCTCT and antisense 5′→3′ATCTACTGCCTGGGGACCTT (XM_039092491); (2) *Gapdh* sense 5′→3′ ACATGGCCTCCAAGGAGTAA and antisense 5′→3′ GGATAGGGCCTCTCTTGCTCA (XM_032902285). The reaction mixture contained 5 µL SybrGreen PCR Master Mix (Ampplied Biosystems, Foster City, CA, USA), 0.4 µL primers, 4 µL DPEC water, and 1 µL cDNA. The amplification was performed on an Applied Biosystems PCR system, with an initial denaturation at 95 °C for 3 s, followed by 40 cycles of denaturation at 95 °C for 3 s, and annealing at 60 °C for 30 s. A melt curve analysis was performed to assess the quality of the amplification. A negative control was included by substituting cDNA with RNase-free water. No amplification of fragments occurred in control samples without reverse transcriptase. Gene expression was quantified using the 2^−ΔΔCt^ method [35]. All reactions were performed in duplicate, and relative expression levels were compared between experimental groups.

### 2.12. Determination of ROS Content

The ROS content was assessed by measuring the conversion of 2′,7′-dichlorodihydrofluorescin diacetate (DCFH-DA-D6883 Sigma^®^) to the fluorescent dichlorofluorescein (DCF) induced by ROS [36]. Briefly, frozen cardiac tissue samples were sectioned at 15 μm thickness using a cryostat (Slee Mainz^®^) and placed in 6-well plates containing 1X PBS. The sections were then transferred to microscope slides and exposed to a fluorescent probe (30 µL per section) at a concentration of 50 μM, followed by incubation for 20 min at 37 °C. The conversion of DCFH to DCF was analyzed using a fluorescence microscope (Olympus^®^ BX53F) with a filter set to wavelengths between 492 and 495 nm. Images were quickly captured with a 10× objective at 0, 2, and 4 min time intervals. The results were expressed as the area under the fluorescence decay curve [37].

### 2.13. Western Blot

Heart muscle samples (~100 mg) were homogenized in cold lysis buffer (1% Triton X-100, 100 mM Tris pH 8.0, 20% glycerol, 0.2 mM EDTA) containing a protease inhibitor cocktail (cOmplete™ Protease Inhibitor Cocktail, Roche, Mannheim, Germany) and a phosphatase inhibitor cocktail (Phosphatase Inhibitor Cocktail PhosStop, Roche, Germany). The lysates were centrifuged at 12,000× *g* for 5 min at 4 °C, and the supernatant was collected. The protein concentration was assessed using the Bradford method [38], with bovine serum albumin (BSA) as the standard. The samples were then diluted in a sample buffer and heated at 100 °C for 5 min. Approximately 40 µg of total protein was separated on a 12% polyacrylamide gel using SDS-PAGE (Bio-Rad, Mini-Protean Tetra Cell System, Hercules, CA, USA) before being transferred to PVDF membranes (Bio-Rad, Power Pac Basic Power Supply, Hercules, CA, USA). To assess transfer efficiency, the membranes were stained with Ponceau. Membranes were blocked by incubation in 5% BSA in TBS-T buffer for 1 h at room temperature. They were then incubated with the primary antibody (1:1000 dilution in 1% BSA, TBS-T) under agitation at 4 °C overnight. Following this, the membranes were incubated with an HRP-conjugated secondary antibody (1:2000 dilution in 3% BSA, TBS-T) for 2 h at room temperature. The primary antibodies used were anti-Sod2 (#MAB3419, R&D Systems, ~22 kDa) and anti-Gapdh (#2118, Cell Signaling, ~37 kDa). The secondary antibodies used were anti-rabbit IgG and anti-mouse IgG (#7074S and #7076S, respectively, both from Cell Signaling, Danvers, MA, USA). PageRuler Plus Prestained Protein Lader, 10 to 250 kDa, Thermo Fisher (#26619) was used as an indicator of molecular weight. Membranes were incubated with Luminata Forte (Merck Millipore, Darmstadt, Germany) for 3 min, protected from light. Bands were visualized by photodocumentation (Loccus Biotecnologia, Cotia, Brazil) and quantified using ImageJ software. The relative content values are presented as arbitrary units.

### 2.14. Statistical Analysis

Data were normally distributed, as confirmed by the Kolmogorov–Smirnov test, and are presented as mean ± standard deviation. Differences between groups were analyzed using the Student’s *t*-test or two-way ANOVA followed by Tukey’s post hoc test, using GraphPad Prism software (Version 8, San Diego, CA, USA). For sample size calculation, we used the contractility index data (mean and standard deviation) from the control and calorie-restricted groups, as reported by Rodrigues et al. [29]. A power of 92% and an alpha error of 5% were considered, reaching 11 rats per group. A *p*-value of <0.05 was considered statistically significant for all analyses.

## 3. Results

### 3.1. Experimental Model and Cardiovascular Risk Factors

Initially, we observed that the body weight of offspring in the R group was significantly lower than that of the C group from the first weeks of lactation, and this difference was maintained until the 12th week (Table S1). After ovariectomy, the animals exhibited a greater weight gain compared to the sham groups (C-Sham and R-Sham) (Table 1). As expected, the CR animals had a lower final body weight than the control group, with the restricted animals showing a 36.2% reduction in final weight compared to the Sham animals. Similarly, the C-OVX group exhibited a higher final weight compared to the R-OVX group. When comparing the C-Sham group with the C-OVX group, a 15.7% weight gain was observed in the castrated group, confirming the weight gain associated with this phase. Body weight was affected by both CR and ovariectomy, showing an interaction between these two factors.

The efficacy of the ovariectomy was confirmed by the reduction in uterine mass and E2 levels in both the control and restricted animals (Table 1) [39]. Importantly, E2 levels were similar in both the R-Sham and C-Sham groups, indicating that this intervention did not affect ovarian hormone production.

The dietary influence on tissue weight was observed in nearly all tissues analyzed, with the exception of the heart and left ventricle (when normalized), suggesting that the weight of these tissues was not affected by the diet. CR also led to reduced liver weight and significantly lower retroperitoneal and parametrial fat weight, both before and after body weight correction. These fat reductions were at least 70% lower compared to controls, and this was accompanied by a reduction in overall body weight in the restricted animals. Regarding ovariectomy, it affected the relative weight of the hearts, left ventricles, and liver (both absolute and relative).

**Figure 1:**
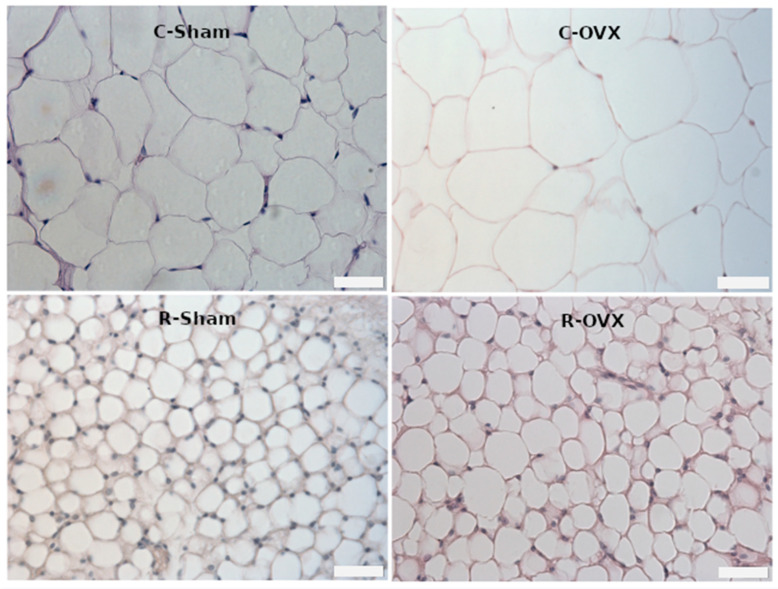
Histological sections of retroperitoneal adipose tissue stained with hematoxylin and eosin. Scale bar, 40 µm; C-Sham: Control rats (ad libitum) without ovariectomy; C-OVX: Ovariectomized control rats (ad libitum); R-Sham: Rats with caloric restriction without ovariectomy; R-OVX: Ovariectomized rats with caloric restriction.

Table 2 shows the effects of 50% CR on major cardiovascular risk factors. The results indicated that CR significantly reduced most of these risk factors. Compared to the control groups, animals subjected to CR demonstrated improvements in plasma lipid profiles, evidenced by reductions in total cholesterol and LDL levels. Additionally, the restricted animals exhibited improved glucose tolerance and insulin response, indicating increased insulin sensitivity. Cardiovascular parameters also showed the protective effects of CR, including reduced systolic blood pressure (SBP), lower heart rate (HR), and decreased cardiac workload, as indicated by a reduced double product (DP). Furthermore, restricted animals had smaller retroperitoneal fat weights, with approximately 80% less fat, as shown by a smaller adipocyte area (Table 2 and Figure 1). These data collectively demonstrate that CR reduces the risk of developing cardiometabolic diseases.

### 3.2. Cardiac Function, Mitochondrial Content and ROS Production

**Figure 2:**
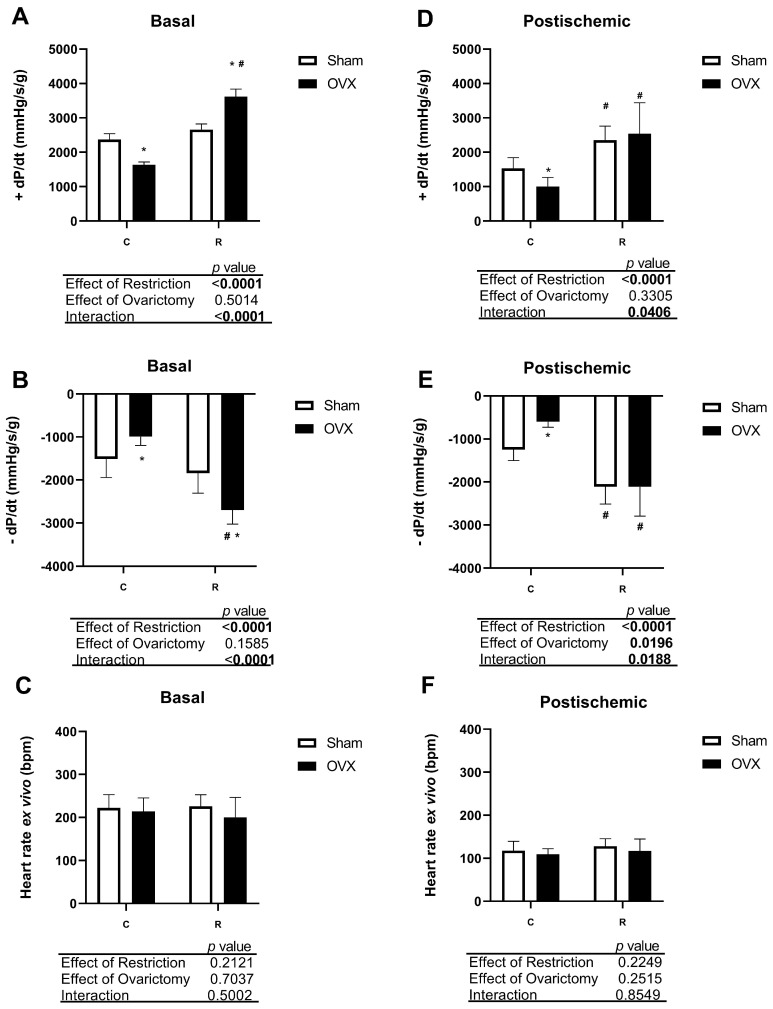
Basal and post-ischemic cardiac function in isolated hearts using the Langendorff technique. (**A**) Basal contractility index (+dP/dt). (**B**) Basal relaxation index (−dP/dt). (**C**) Basal heart rate basal. (**D**) Post-ischemic contractility index (+dP/dt). (**E**) Post-ischemic relaxation index (−dP/dt). (**F**) Post-ischemic heart rate basal index. Significant differences were determined by two-way ANOVA followed by Tukey’s post hoc test (*p* < 0.05, *n* = 10–11/group); * denotes significant difference between ovariectomized rats (C-OVX or R-OVX) and their respective Sham groups (C-Sham or R-Sham); # denotes significant difference between rats with caloric restriction (R-Sham or R-OVX) and their respective control groups (C-Sham or C-OVX). C-Sham: Control rats (ad libitum) without ovariectomy; C-OVX: Ovariectomized control rats (ad libitum); R-Sham: Rats with caloric restriction without ovariectomy; R-OVX: Ovariectomized rats with caloric restriction.

To evaluate cardiac function without neuro-hormonal influence, we used the Langendorff isolated heart technique. Initially, we observed basal cardiac function results similar to those of our previous study [29], where Rodrigues et al. (2021) demonstrated that ICR from birth prevents ovarian castration-induced cardiac dysfunction in adult rats. Based on this, we confirmed an increase in contractility and relaxation of the hearts in the restricted animals (C-Sham vs. R-Sham) and prevention of ovariectomy-induced effects (R-Sham vs. R-OVX) (Figure 2A,B). Ex vivo HR was similar across all groups (Figure 2C).

The cardioprotective effects of CR were also evaluated in response to cardiac injury, where isolated hearts were subjected to 10 min of global ischemia followed by 15 min of reperfusion. As described in previous studies with ovariectomized rats [11,40,41], we observed a deleterious effect of ischemia in the castrated animals (Figure 2D–F). In contrast, the restricted animals exhibited better cardiac function post-ischemia, with higher contraction and relaxation efficiency. HR did not show significant differences between the groups. As expected, there was a reduction in cardiac function following cardiac stress in all groups; however, it was noted that ±dP/dt post-ischemic indices in the hearts of the CR animals showed values close to those observed in control hearts at baseline, without ischemia.

**Figure 3:**
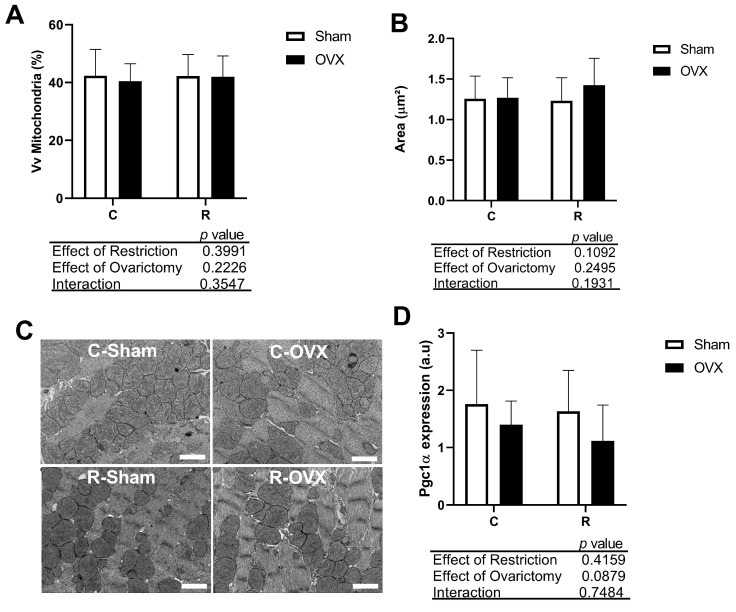
Mitochondrial content in cardiac tissue. (**A**) Volumetric density (Vv) of normal mitochondria. (**B**) Mitochondrial area. (**C**) Transmission electron micrographs of cross-sectional images of the post-ischemic left ventricle (ultrastructural view) from different experimental groups. (**D**) *Pgc1-α* expression. No difference by two-way ANOVA, *n* = 72 cells/group; Scale bar, 2.0 µm. C-Sham: Control rats (ad libitum) without ovariectomy; C-OVX: Ovariectomized control rats (ad libitum); R-Sham: Rats with caloric restriction without ovariectomy; R-OVX: Ovariectomized rats with caloric restriction.

To better understand the cardioprotective effects of our CR protocol in I/R injury, we assessed the mitochondrial ultrastructure in post-ischemic hearts. As shown in Figure 3, we did not observe differences in the volumetric density and mitochondria area between the groups, indicating that neither CR nor ovariectomy interfered with mitochondrial biogenesis in cardiomyocytes. This was further confirmed by the gene expression (mRNA) analysis of *Pgc-1α* in cardiac tissue using real-time PCR (Figure 3D), as *Pgc-1α* is a transcriptional factor involved in mitochondrial biogenesis, and in our study, no differences were observed between the groups (Figure 3D).

**Figure 4:**
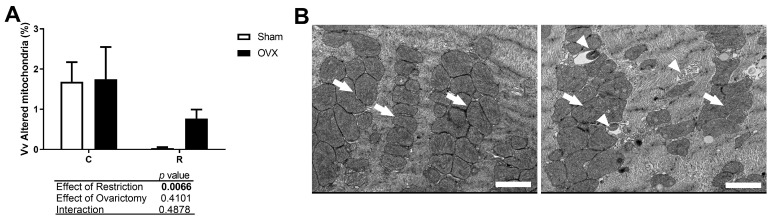
Transmission electron microscopy analysis of the morphology of altered mitochondria in cardiac tissue. (**A**) Volumetric density (Vv) of altered mitochondria. (**B**) Transmission electron micrographs of cross-sectional images of the post-ischemic left ventricle (ultrastructural view), with representation of normal mitochondria (arrows) from R-Sham rat (left image) and altered mitochondria (arrowheads) from a C-OVX rat (right image), showing vacuolization, pleomorphism, and disordered and few cristae. Scale bar, 2.0 µm. Two-way ANOVA followed by Tukey’s post hoc test, *n* = 72 cells/group; C-Sham: Control rats (ad libitum) without ovariectomy; C-OVX: Ovariectomized control rats (ad libitum); R-Sham: Rats with caloric restriction without ovariectomy; R-OVX: Ovariectomized rats with caloric restriction.

Although no differences in volumetric mitochondrial density were observed for normal mitochondria, the restricted animals showed a reduction in the volumetric density of altered mitochondria (Figure 4A). The mitochondrial alterations observed included vacuolization, pleomorphism, and disordered and fewer cristae. In contrast, normal mitochondria were electrodense with well-defined cristae (Figure 4B).

**Figure 5:**
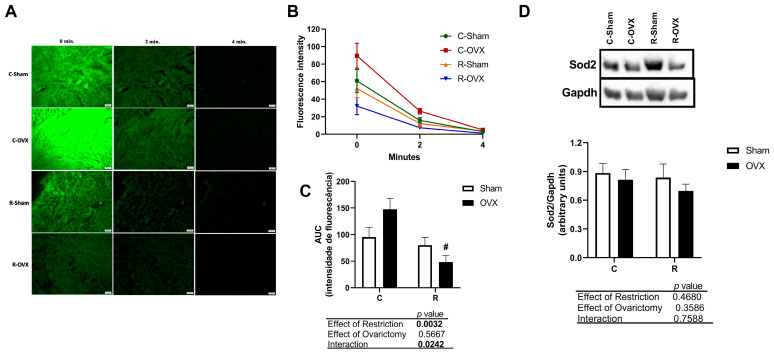
Effects of intense caloric restriction on reactive oxygen species production by DCFH-DA in cardiac tissue. (**A**) ROS production evaluated by fluorescence microscopy images associated with DCFH-DA, Scale bar, 100 µm. (**B**,**C**) Fluorescence intensity at 0, 2, and 4 min. (**D**) Sod2 content; # denotes significant difference between C-OVX or R-OVX; in two-way ANOVA followed by Tukey’s post hoc test, *n* = 6–7/group; C-Sham: Control rats (ad libitum) without ovariectomy; C-OVX: Ovariectomized control rats (ad libitum); R-Sham: Rats with caloric restriction without ovariectomy; R-OVX: Ovariectomized rats with caloric restriction. Significant differences were determined by two-way ANOVA followed by Tukey’s post hoc test.

As demonstrated, there was a reduction in the number of altered mitochondria in the CR animals, and since no differences were observed in mitochondrial biogenesis, we proceeded to analyze ROS production in cardiac tissues using the fluorescent probe DCFH-DA. We found that animals on the diet showed reduced ROS generation, as demonstrated by the shorter fluorescence decay time at 0, 2, and 4 min (Figure 5A). At time 0, following radiation excitation, the post-ischemic cardiac tissues of the restricted animals exhibited lower fluorescence intensity, with approximately 60% less fluorescence when comparing the castrated groups (C-OVX vs. R-OVX) (Figure 5B,C). We also evaluated the Sod2 content, which showed no differences between the groups (Figure 5D).

## 4. Discussion

This study aimed to investigate whether intense caloric restriction (ICR) from birth exerts a cardioprotective effect against the deleterious consequences of reduced estrogen levels. To test this hypothesis, we employed ovariectomy as an experimental model of estrogen deprivation. Our results provide compelling evidence that ICR from birth not only reduces key cardiometabolic risk factors but also improves both basal and post-ischemic cardiac function in ovariectomized rats, effectively mitigating the harmful effects of estrogen reduction. Notably, restricted animals exhibited increased resistance to ischemia and fewer mitochondrial alterations, even following ovariectomy. These protective effects appear to be linked to an improved oxidative profile, as we observed a significant reduction in ROS in the cardiomyocytes of CR-treated animals.

Initially, we monitored the weekly body weight of the animals. From the first week of life, we observed a significant difference in body weight, with restricted animals growing less than those fed ad libitum. Studies show that ICR leads to a significant reduction in the weight of rodents. Despite these notable changes, CR does not have negative health effects on the animals, and it is probable that they exhibit metabolic adaptations that appear to counterbalance possible negative impacts [42,43]. Despite the reduced body weight in the CR group, organ weights were preserved, likely due to metabolic reprogramming in the restricted animals, which develop adaptive mechanisms to prevent malnutrition, altering how energy is stored and utilized in the body [44]. The heart and left ventricle weights, normalized to body weight, were not influenced by diet, indicating that CR did not affect the size of these tissues. These results align with previous findings from our group, where ICR preserved the weight of these tissues [29]. Moreover, ICR induced beneficial changes in the heart’s structure, improving contractile function [26].

Next, we assessed the effects of ICR on key cardiovascular risk factors. The results demonstrated that ICR significantly reduced most of the risk factors, supporting the literature that links intense and moderate caloric restriction (in both humans and animals) to reductions in adipose tissue, triglycerides, and total cholesterol [45,46,47]. Additionally, a long-term severe caloric restriction has a beneficial effect against the risk of developing cardiovascular diseases, evidenced by reductions in total cholesterol, LDL, triglycerides, and cardiac arrhythmia [28]. In our study, we observed a reduction in HDL cholesterol in the restricted animals, but this may be explained by the fact that these animals had lower amounts of adipose tissue [48].

The restricted animals also exhibited improved glucose tolerance and insulin response, indicating greater insulin sensitivity. Studies show that CR regulates insulin sensitivity and glucose homeostasis in humans [49,50] and rodents [50,51,52,53]. CR also reduced blood glucose levels in diabetic mice, improving glucose homeostasis [54].

When evaluating the effect of ovariectomy, we observed an increase in SBP and HR in these animals (C-OVX). This effect can be explained by the abrupt reduction in estrogen levels [55], as the absence of these hormones can lead to vascular aging, arterial stiffness, obesity, altered insulin sensitivity, oxidative stress, increased cholesterol, among other risk factors [56,57]. Additionally, the administration of E2 to ovariectomized rats prone to hypertension regulated SBP, preventing hypertension [58].

When evaluating in vivo SBP and HR in the restricted animals, we observed a reduction in both parameters compared to control animals. R-OVX exhibited SBP values similar to the non-castrated control group (C-Sham), highlighting the beneficial effects of this intervention on SBP. CR was also effective in reducing HR, showing lower values in these groups compared to their controls. In agreement with our findings, 40% CR reduced blood pressure in obese and hypertensive animals [59]. Other studies have also demonstrated the protective effect of caloric restriction on BP and HR in rats [60,61].

From the values of blood pressure and HR, we calculated the DP, a predictor of cardiac overload in humans and animals [62], as an indicator of cardiac workload. We observed in our study that CR was beneficial in reducing DP values, especially when compared to the C-OVX group, which showed greater cardiac effort, evidenced by the elevated index value. Based on these findings, it can be inferred that CR provided cardioprotection by reducing SBP and HR, ultimately leading to less cardiac overload, as indicated by lower DP.

The beneficial effect of CR on cardiac function in the absence of estrogen was also observed through an isolated heart analysis using the Langendorff technique. We observed an improvement in baseline cardiac function in the restricted animals, supporting our previous study [29]. Given this, we evaluated the effects of CR on cardiac function after injury. I/R leads to the dysfunction of myocardial energy metabolism and ATP deficiency, causing a variety of injuries, such as the dysfunction of the cation pump, calcium overload within cells, and increased production of ROS [63]. In our study, the post-ischemic function of the hearts from the restricted animals was significantly better compared to ad libitum control animals. These findings are supported by other studies showing that long-term CR protects cardiac function during the post-ischemic period in rats [64,65]. Melo et al. (2026) showed that 50% CR from birth resulted in a 60% greater post-ischemic cardiac function compared to the ad libitum group [27]. In another study, Guo et al. (2023) demonstrated that 70% CR significantly reduced myocardial injury induced by I/R in elderly mice, as evidenced by a reduction in infarct size [66].

The heart rate (HR) data from the isolated hearts showed no significant difference, mirroring the findings observed in vivo. This suggests that one of the beneficial effects of caloric restriction (CR) on the heart is exerted directly on the organ’s contractile function, independent of neuro-hormonal stimulation and relying solely on the sinus node for electrical conduction [67]. In vivo, the reduction in HR due to CR may result from an increased parasympathetic tone, which enhances the activity of cholinergic cardiovagal neurons in the brainstem [61].

One of the mechanisms involved in cardioprotection, particularly when subjected to I/R, is the alteration of the mitochondrial ultrastructure. CR might promote adaptive reprogramming in response to cardiac insults by altering mitochondrial number and function. However, in the present study, we did not observe differences in mitochondrial volumetric densities or in *Pgc-1α* gene expression between the groups, indicating that CR did not interfere with mitochondrial biogenesis in cardiomyocytes.

In the literature, Hancock et al. (2011) also did not observe differences in gene expression in restricted animals, suggesting that CR protects mitochondria from aging, not by stimulating mitochondrial biogenesis but by protecting against DNA damage, enzyme abnormalities, and mitochondrial loss [68]. In another study, Miller et al. also showed that 40% CR in mice does not increase mitochondrial protein synthesis but maintains it at levels similar to those of ad libitum animals [69], demonstrating that the fact that CR increases mitochondrial biogenesis is paradoxical, given that this process is energetically demanding (protein synthesis), which would be counterproductive during a state of energy scarcity, as occurs in CR. These results are similar to our findings, as we did not observe changes in biogenesis but showed that the animals subjected to CR had a reduction in the number of damaged mitochondria, suggesting that the lower basal metabolic rate promoted by CR may be leading to cardioprotection, probably through other mitochondrial protection pathways.

Given the greater resistance to ischemia and the reduction of damaged mitochondria in the restricted animals, even after ovariectomy, we assessed the ROS in cardiomyocytes and observed an improvement in the oxidative profile, as evidenced by reduced fluorescence. It is important to highlight that mitochondria, in addition to generating ATP, play key roles in cell survival and death [70]. Mitochondrial dynamics, through fusion and fission processes, are essential to maintain organelle integrity, as they facilitate replication, repair, and the elimination of defective mitochondria via mitophagy, ensuring the health of the mitochondrial network for normal cell function [71]. Mitochondria are also the primary cellular sources of free radicals, a process exacerbated by ovariectomy and ischemic events such as ischemia/reperfusion (I/R). During reproductive years, estrogens mediate antioxidant actions, promoting a vasoprotective effect by increasing the expression of enzymes like superoxide dismutase (SOD) and catalase (CAT). Thus, the reduction in ovarian hormones, as seen in ovariectomy, creates an oxidative stress environment harmful to the heart [72,73,74]. Similarly, in I/R injury, ROS are generated at high levels, leading to deleterious effects on cardiac function [75].

In light of this, CR is reported to have positive effects in reducing oxidative stress [76,77,78], showing benefits for cardiac tissue in long-term restriction protocols [79,80]. In addition to reducing ROS production, CR may improve the oxidative profile by modifying mitochondrial membranes, making them less prone to damage. Furthermore, animals subjected to CR also modulate their antioxidant defenses to more efficiently remove free radicals generated [43,81]. This can be explained by the prolonged fasting state of CR animals, which favors autophagy and maintains cell integrity. Thus, restriction reduces the deleterious effects of oxidative stress and contributes to the renewal and recycling of cellular components [82,83]. A long-term CR also attenuates myocardial oxidative damage after I/R at the mitochondrial level during reperfusion [84].

CR likely exerts its cardioprotective effects through multiple cellular signaling pathways. One possible mechanism involves the modulation of NAD+ levels and the NAD+/NADH ratio in the heart. NAD+ and NADH are critical coenzymes in redox reactions that regulate cardiac function, including mitochondrial β-oxidation and oxidative phosphorylation. CR has been shown to elevate NAD+ levels in the heart, enhancing energy production and supporting cardiovascular health [85]. NAD+ also serves as an essential cofactor for enzymes such as sirtuins, particularly Sirtuin 1 (Sirt1), which protects cardiomyocytes against damage from ROS and I/R injury [85,86]. Sirtuins are a conserved family of NAD+-dependent enzymes (SIRT1 to SIRT7) that regulate diverse cellular processes. Nuclear sirtuins (SIRT1, SIRT6, SIRT7) modulate gene expression, mitochondrial sirtuins (SIRT3, SIRT4, SIRT5) influence cellular metabolism and oxidative stress, and cytoplasmic SIRT2 plays roles in DNA repair and apoptosis [87]. CR has been associated with an increased expression of sirtuins [88,89,90,91], with Sirt1 playing a pivotal role in maintaining cardiac mitochondrial integrity [92].

NAD+ and NADH also influence AMP-activated protein kinase (AMPK), a key energy-sensing enzyme that maintains cellular energy homeostasis [93]. CR has been reported to increase NAD+ levels [94] and activate sirtuins in cardiac muscle [66,95,96]. The activation of Sirt1 has been implicated in the cardioprotective effects of CR by suppressing local complement system activation following I/R injury [88]. Additionally, AMPK activation, another proposed pathway, has been shown to enhance NAD+ levels and activate sirtuins [66], while also interacting with the PI3K/Akt pathway. The PI3K/Akt pathway has been linked to improved mitochondrial fusion, increased cardiomyocyte resilience to ischemia, and enhanced cell survival [97]. CR has also been proposed to act as an antioxidant defense mechanism in rodent hearts by increasing SOD2 protein levels [28]. Our previous findings demonstrated enhanced cardiac SOD activity in restricted animals [29]. However, in the present study, neither CR nor ovariectomy altered SOD content. This does not preclude the possibility that CR enhances SOD activity through post-translational modifications or signaling cascades, rather than increasing its cellular abundance.

The limitation of this study is the absence of functional assessments of mitochondrial bioenergetics or a detailed analysis of cellular signaling pathways upregulated or downregulated by CR. While the literature highlights potential mechanisms underlying CR-induced cardioprotection, this study focused on mitochondrial ultrastructural changes and ROS content reduction. Future studies are required to explore these signaling pathways in greater depth and to elucidate how lifelong CR contributes to mitochondrial function and ROS mitigation. Thus, we speculate that CR protects the heart in the absence of estrogenic protection by preserving mitochondrial function and reducing mitochondrial oxidative production, potentially in combination with enhanced endogenous antioxidant activity, without altering mitochondrial biogenesis.

## 5. Conclusions

In conclusion, our findings revealed that lifelong caloric restriction (CR) exerts positive effects on cardiac function and reduces key cardiometabolic risk factors. Additionally, we demonstrated that CR protects the heart from ischemia/reperfusion injuries, likely by reducing ROS, thereby mitigating mitochondrial damage caused by ovariectomy and I/R.

## Figures and Tables

**Table 1:** Characterization of experimental groups at 120 days of age.

	Groups				*p* Values		
	C-Sham	C-OVX	R-Sham	R-OVX	Effect of ICR	Effect of OVX	Interaction
Body weight (g)	257.5 ± 14.95	305.0 ± 15.09	164.5 ± 18.17 #	186.5 ± 18.27 *#	**<0.0001**	**<0.0001**	**0.0210**
Heart (g)	1.06 ± 0.08	1.12 ± 0.11	0.65 ± 0.07 #	0.67 ± 0.07 #	**<0.0001**	0.2074	0.5607
Heart (mg/g)	4.13 ±0.25	3.67 ± 0.40	3.99 ±0.52	3.62 ± 0.40	0.4753	**0.0024**	0.7274
Left ventricle (g)	0.78 ± 0.05	0.81 ± 0.07	0.50 ± 0.05 #	0.51 ± 0.03 #	**<0.0001**	0.1991	0.6215
Left ventricle (mg/g)	3.02 ± 0.14	2.66 ± 0.28 *	3.05 ±0.35	2.77 ± 0.27 *	0.4205	**0.0006**	0.6354
Liver (g)	7.91 ± 0.70	8.80 ± 0.82 *	4.35 ± 0.66 #	4.58 ± 0.52 #	**<0.0001**	**0.0151**	0.1455
Liver (mg/g)	30.73 ± 2.16	28.82 ± 1.96	26.41 ± 2.39 #	24.54 ± 0.92 #	**<0.0001**	**0.0040**	0.9742
Retroperitoneal fat (g)	2.71 ± 0.54	3.17 ± 0.69	0.34 ± 0.16 #	0.31 ± 0.19 #	**<0.0001**	0.1548	0.1001
Retroperitoneal fat (mg/g)	10.57 ± 2.21	10.36 ± 2.09	2.08 ± 0.97 #	1.61 ± 0.92 #	**<0.0001**	0.5170	0.8088
Parametrial fat (g)	4.00 ± 0.95	4.05 ± 0.78	0.69 ± 0.25 #	0.66 ± 0.43 #	**<0.0001**	0.9699	0.8462
Parametrial fat (mg/g)	15.45 ± 3.24	13.24 ± 2.28	4.24 ± 1.68 #	3.40 ± 2.06 #	**<0.0001**	0.0503	0.3671
Uterus (g)	0.54 ± 0.16	0.23 ± 0.09 *	0.15 ± 0.04 #	0.06 ± 0.02 *#	**<0.0001**	**<0.0001**	**0.0006**
Uterus (mg/g)	2.11 ± 0.61	0.78 ± 0.36 *	1.02 ± 0.29 #	0.39 ± 0.13 *#	**<0.0001**	**<0.0001**	**0.0043**
17β-Estradiol (pg/dL)	46.26 ± 27.45	9.02 ± 8.32 *	54.73 ± 37.64	17.15 ± 8.70 *	**0.9856**	**0.0004**	0.3704

* Significant difference in ovariectomy (OVX) compared to its corresponding Sham group (Control or Restricted). # Significant difference in caloric restriction compared to its corresponding control group (Sham or OVX) (*p* < 0.05, two-way ANOVA followed by post hoc Tukey test, *n* = 10–11/group). Relative weight (mg/g): [organ weight (g)/body weight (g)] × 1000; C-Sham: Control rats (ad libitum) without ovariectomy; C-OVX: Ovariectomized control rats (ad libitum); R-Sham: Rats with caloric restriction without ovariectomy; R-OVX: Ovariectomized rats with caloric restriction.

**Table 2:** Cardiometabolic risk factors.

	Groups				*p* Values		
	C-Sham	C-OVX	R-Sham	R-OVX	Effect of ICR	Effect of OVX	Interaction
Total cholesterol (mg/dL)	57.5 ±13.6	100.5 ± 13.9 *	56.5 ± 5.7	74.4 ± 7.2 *#	**0.0060**	**<0.0001**	**0.0098**
HDL cholesterol (mg/dL)	22.8 ± 4.8	31.2 ± 3.6 *	21.2 ± 2.2	24.4 ± 3.1 #	**0.0093**	**0.0008**	0.0986
LDL cholesterol (mg/dL)	20.7 ± 12.4	54.4 ± 8.9 *	17.4 ± 8.2	33.2 ± 7.1 *#	**0.0045**	**<0.0001**	**0.0299**
VLDL cholesterol (mg/dL))	13.9 ± 4.5	14.8 ± 5.1	17.9 ± 3.9	16.7 ± 2.4	0.0938	0.9487	0.5358
Triglycerides (mg/dL)	69.5 ± 22.5	74.3 ± 25.5	89.7 ± 19.7	83.8 ± 12.4	0.0938	0.9487	0.5358
Basal blood glucose (mg/dL)	105.3 ± 7.8	104.7 ± 11.2	92.1 ± 10.5.1	92.4 ± 6.0	**0.0015**	0.9628	0.8992
OGTT (Area under the curve)	18,452 ± 2045	16,786 ± 1124	15,044 ± 1354 #	14,607 ± 1622	**0.0003**	0.1175	0.3501
IITT (Area under the curve)	4177 ± 532	4247 ± 586	3887 ± 415	3500 ± 304 #	**0.0109**	0.4028	0.2319
Systolic blood pressure (mmHg)	113.7± 7.9	131.2 ± 12.3 *	109.2 ± 6.8	116.4 ± 8.87 #	**0.0024**	**0.0028**	0.4071
Heart rate in vivo (bpm)	359 ±26	396 ± 36.8 *	337 ± 18.5	358 ± 31.9 #	**0.0084**	**0.0010**	0.1996
Double product (mmHg * bpm)	40,787.5 ± 3526	52,054 ± 7032 *	32,088 ± 7535	41,595 ± 3475 #	**0.0002**	**0.0002**	0.6112
Adipocyte area (μm^2^)	4999 ± 1306	6228.3 ± * 1632	1105 ± 614 #	1734 ± 486 #	**<0.0001**	**<0.0001**	0.0955

* Significant difference in ovariectomy (OVX) compared to its corresponding Sham group (C-Sham vs. C-OVX or R-Sham vs. R-OVX). # Significant difference in caloric restriction compared to its corresponding control group (R-Sham vs. C-Sham or R-OVX vs. C-OVX) (*p* < 0.05, two-way ANOVA followed by post hoc Tukey test, *n* = 6–7/group). C-Sham: Control rats (ad libitum) without ovariectomy; C-OVX: Ovariectomized control rats (ad libitum); R-Sham: Rats with caloric restriction without ovariectomy; R-OVX: Ovariectomized rats with caloric restriction.

## Data Availability

Data are contained within the article and Supplementary Materials. Further inquiries can be directed to the corresponding author.

## Supplementary Materials

The following supporting information can be downloaded at: https://www.mdpi.com/article/10.3390/antiox14020169/s1, Table S1: Body weight.

## Nomenclature

The following abbreviations are used in this manuscript:

AMPK	AMP-activated protein kinase
Akt	Protein kinase B
AUC	Area under the curve
BSA	Bovine serum albumin
C	Control group
CAT	Catalase
CEUA	Animal Ethics Committee
C-OVX	Ovariectomized control rats (ad libitum)
C-Sham	Control rats (ad libitum) without ovariectomy
CR	Caloric restriction
DCF	Fluorescent dichlorofluorescein
DCFH-DA	2′,7′-dichlorodihydrofluorescin diacetate
dNTP	Deoxynucleotide triphosphate
DP	Double product
DTT	Dithiothreitol
E2	17β-estradiol
EDTA	Ethylenediaminetetraacetic acid
HDL	High-density lipoprotein
HR	Heart rate
HRP	Horseradish peroxidase
HRT	Hormone replacement therapy
IgG	Immunoglobulin G
I/R	Ischemia/Reperfusion
ICR	Intense caloric restriction
IITT	Intraperitoneal Insulin Tolerance Test
NAD+	Nicotinamide adenine dinucleotide
NADH	Nicotinamide adenine dinucleotide (NAD) + hydrogen (H)
OGTT	Oral Glucose Tolerance Test
PBS	Phosphate buffer solution
PCR	Polymerase chain reaction
Pgc-1α	Peroxisome proliferator-activated receptor gamma coactivator 1α
PI3K	Phosphoinositide 3-kinase
PVDF	Polyvinylidene difluoride
R	Restriction group
R-OVX	Rats with caloric restriction ovariectomized
R-Sham	Rats with caloric restriction without ovariectomy
ROS	Reactive oxygen species
SBP	Systolic blood pressure
SDS-PAGE	Sodium dodecyl-sulfate polyacrylamide gel electrophoresis
SIRT	Sirtuin
SOD	Superoxide dismutase
TBS-T	Tris Buffered Saline with Tween
UFVJM	Universidade Federal dos Vales do Jequitinhonha e Mucuri
Vv	Volumetric density

## References

[1] Filho J.F.L., Baccaro L.F.C., Fernandes T., Conde D.M., Costa-Paiva L., Neto A.M.P. (2015). Epidemiologia Da Menopausa e Dos Sintomas Climatéricos Em Mulheres de Uma Região Metropolitana No Sudeste Do Brasil: Inquérito Populacional Domiciliar. Rev. Bras. Ginecol. Obstet..

[2] Landgren B.M., Collins A., Csemiczky G., Burger H.G., Baksheev L., Robertson D.M. (2004). Menopause Transition: Annual Changes in Serum Hormonal Patterns over the Menstrual Cycle in Women during a Nine-Year Period Prior to Menopause. J. Clin. Endocrinol. Metab..

[3] Mauvais-Jarvis F., Clegg D.J., Hevener A.L. (2013). The Role of Estrogens in Control of Energy Balance and Glucose Homeostasis. Endocr. Rev..

[4] Reckelhoff J.F. (2018). Sex Differences in Regulation of Blood Pressure. Adv. Exp. Med. Biol..

[5] Mendelsohn M.E., Karas R.H. (1999). The Protective Effects of Estrogen on the Cardiovascular System. N. Engl. J. Med..

[6] Dosi R., Bhatt N., Shah P., Patell R. (2014). Cardiovascular Disease and Menopause. J. Clin. Diagn. Res..

[7] Debortoli A.R., do Nascimento Rouver W., Delgado N.T.B., Mengal V., Claudio E.R.G., Pernomian L., Bendhack L.M., Moysés M.R., Santos R.L. (2017). dos GPER Modulates Tone and Coronary Vascular Reactivity in Male and Female Rats. J. Mol. Endocrinol..

[8] Santos R.L., Lima J.T., Rouver W.N., Moysés M.R. (2016). Deficiency of Sex Hormones Does Not Affect 17-ß-Estradiol-Induced Coronary Vasodilation in the Isolated Rat Heart. Braz. J. Med. Biol. Res..

[9] Morkuniene R., Arandarcikaite O., Ivanoviene L., Borutaite V. (2010). Estradiol-Induced Protection against Ischemia-Induced Heart Mitochondrial Damage and Caspase Activation Is Mediated by Protein Kinase G. Biochim. Biophys. Acta-Bioenerg..

[10] Brinton R.D. (2012). Minireview: Translational Animal Models of Human Menopause: Challenges and Emerging Opportunities. Endocrinology.

[11] Chae S.-U., Ha K.-C., Piao C.-S., Chae S.-W., Chae H.-J. (2007). Estrogen Attenuates Cardiac Ischemia-Reperfusion Injury via Inhibition of Calpain-Mediated Bid Cleavage. Arch. Pharm. Res..

[12] Wang M., Crisostomo P., Wairiuko G.M., Meldrum D.R. (2006). Estrogen Receptor-α Mediates Acute Myocardial Protection in Females. Am. J. Physiol.-Heart Circ. Physiol..

[13] Morimoto K., Uji M., Ueyama T., Kimura H., Kohno T., Takamata A., Yano S., Yoshida K.-I. (2008). Estrogen Replacement Suppresses Pressor Response and Oxidative Stress Induced by Cage-Switch Stress in Ovariectomized Rats. Ann. N. Y. Acad. Sci..

[14] Newson L. (2018). Menopause and Cardiovascular Disease. Post Reprod. Health.

[15] Liu H., Pedram A., Kim J.K. (2011). Oestrogen Prevents Cardiomyocyte Apoptosis by Suppressing P38α-Mediated Activation of P53 and by down-Regulating P53 Inhibition on P38β. Cardiovasc. Res..

[16] Leuzzi C., Marzullo R., Modena M.G. (2012). La Menopausa è Un Fattore Di Rischio per La Cardiopatia Ischemica?. G. Ital. Cardiol..

[17] Amaral A.G., da Silva C.C.C., Serna J.D.C., Honorato-Sampaio K., Freitas J.A., Duarte-Neto A.N., Bloise A.C., Cassina L., Yoshinaga M.Y., Chaves-Filho A.B. (2022). Disruption of Polycystin-1 Cleavage Leads to Cardiac Metabolic Rewiring in Mice. Biochim. Biophys. Acta BBA-Mol. Basis Dis..

[18] Parlee S.D., Lentz S.I., Mori H., MacDougald O.A. (2014). Quantifying Size and Number of Adipocytes in Adipose Tissue. Methods in Enzymology.

[19] Seki S., Horikoshi K., Takeda H., Izumi T., Nagata A., Okumura H., Taniguchi M., Mochizuki S. (2001). Effects of Sustained Low-Flow Ischemia and Reperfusion on Ca2+ Transients and Contractility in Perfused Rat Hearts. Mol. Cell. Biochem..

[20] Davis S.R., Lambrinoudaki I., Lumsden M., Mishra G.D., Pal L., Rees M., Santoro N., Simoncini T. (2015). Menopause. Nat. Rev. Dis. Primer.

[21] Han X., Ren J. (2010). Caloric Restriction and Heart Function: Is There a Sensible Link?. Acta Pharmacol. Sin..

[22] Sung M.M.Y., Dyck J.R.B. (2012). Age-Related Cardiovascular Disease and the Beneficial Effects of Calorie Restriction. Heart Fail. Rev..

[23] Bruss M.D., Khambatta C.F., Ruby M.A., Aggarwal I., Hellerstein M.K. (2010). Calorie Restriction Increases Fatty Acid Synthesis and Whole Body Fat Oxidation Rates. Am. J. Physiol.-Endocrinol. Metab..

[24] Chen C.N., Liao Y.H., Tsai S.C., Thompson L.D.V. (2019). Age-Dependent Effects of Caloric Restriction on mTOR and Ubiquitin-Proteasome Pathways in Skeletal Muscles. GeroScience.

[25] Forster M.J., Morris P., Sohal R.S. (2003). Genotype and Age Influence the Effect of Caloric Intake on Mortality in Mice. FASEB J. Off. Publ. Fed. Am. Soc. Exp. Biol..

[26] Melo D.S., Riul T.R., Esteves E.A., Moraes P.L., Gavioli M., Ferreira F.O., Alves M.N.M., Almeida P.W.M., Guatimosim S., Ferreira A.J. (2013). Effects of Severe Caloric Restriction from Birth on the Hearts of Adult Rats. Appl. Physiol. Nutr. Metab..

[27] Melo D.S., Costa-Pereira L.V., Santos C.S., Mendes B.F., Costa K.B., Santos C.F.F., Rocha-Vieira E., Magalhães F.C., Esteves E.A., Ferreira A.J. (2016). Severe Calorie Restriction Reduces Cardiometabolic Risk Factors and Protects Rat Hearts from Ischemia/Reperfusion Injury. Front. Physiol..

[28] Melo D.D.S., Costa Pereira L., Santos C.S., Mendes B.F., Konig I.F.M., Garcia B.C.C., Queiroz I.P., Moreno L.G., Cassilhas R.C., Esteves E.A. (2023). Intense Caloric Restriction from Birth Prevents Cardiovascular Aging in Rats. Rejuvenation Res..

[29] Rodrigues C.M., Domingues T.E., de Sousa Santos C., Costa-Pereira L.V., Mendes B.F., dos Santos J.M., Costa K.B., Silva G., Cantuária V.L., Rocha-Vieira E. (2021). Cardioprotective Effects of Severe Calorie Restriction from Birth in Adult Ovariectomized Rats. Life Sci..

[30] National Research Council (US) Committee for the Update of the Guide for the Care and Use of Laboratory Animals (2011). Guide for the Care and Use of Laboratory Animals.

[31] Holehan A.M., Merry B.J. (1985). The control of puberty in the dietary restricted female rat. Mech. Ageing Dev..

[32] Depre C., Hue L. (1997). Inhibition of Glycogenolysis by a Glucose Analogue in the Working Rat Heart. J. Mol. Cell. Cardiol..

[33] Voogd A., Sluiter W., Koster J.F. (1991). Contradictory Effects of Superoxide Dismutase after Global or Regional Ischemia in the Isolated Rat Heart. Free Radic. Biol. Med..

[34] Barbosa de Queiroz K., Honorato-Sampaio K., Rossoni Júnior J.V., Andrade Leal D., Pinto A.B.G., Kappes-Becker L., Evangelista E.A., Guerra-Sá R. (2017). Physical Activity Prevents Alterations in Mitochondrial Ultrastructure and Glucometabolic Parameters in a High-Sugar Diet Model. PLoS ONE.

[35] Livak K.J., Schmittgen T.D. (2001). Analysis of Relative Gene Expression Data Using Real-Time Quantitative PCR and the 2^−ΔΔCT^ Method. Methods.

[36] Wang H., Joseph J.A. (1999). Quantifying Cellular Oxidative Stress by Dichlorofluorescein Assay Using Microplate reader11Mention of a Trade Name, Proprietary Product, or Specific Equipment Does Not Constitute a Guarantee by the United States Department of Agriculture and Does Not Imply Its Approval to the Exclusion of Other Products That May Be Suitable. Free Radic. Biol. Med..

[37] Yao Q., Zou X., Liu S., Wu H., Shen Q., Kang J. (2022). Oxidative Stress as a Contributor to Insulin Resistance in the Skeletal Muscles of Mice with Polycystic Ovary Syndrome. Int. J. Mol. Sci..

[38] Bradford M.M. (1976). A Rapid and Sensitive Method for the Quantitation of Microgram Quantities of Protein Utilizing the Principle of Protein-Dye Binding. Anal. Biochem..

[39] Al-Dhubiab B.E., Patel S.S., Morsy M.A., Duvva H., Nair A.B., Deb P.K., Shah J. (2020). The Beneficial Effect of Boswellic Acid on Bone Metabolism and Possible Mechanisms of Action in Experimental Osteoporosis. Nutrients.

[40] Kolodgie F.D., Farb A., Litovsky S.H., Narula J., Jeffers L.A., Lee S.J., Virmani R. (1997). Myocardial Protection of Contractile Function after Global Ischemia by Physiologic Estrogen Replacement in the Ovariectomized Rat. J. Mol. Cell. Cardiol..

[41] Morra E.A., Rodrigues P.L., de Jesus I.C.G., Do Val Lima P.R., Ávila R.A., Zanardo T.É.C., Nogueira B.V., Bers D.M., Guatimosim S., Stefanon I. (2019). Endurance Training Restores Spatially Distinct Cardiac Mitochondrial Function and Myocardial Contractility in Ovariectomized Rats. Free Radic. Biol. Med..

[42] Fontana L., Partridge L. (2015). Promoting Health and Longevity through Diet: From Model Organisms to Humans. Cell.

[43] Speakman J.R., Mitchell S.E. (2011). Caloric Restriction. Mol. Asp. Med..

[44] Anderson R.M., Weindruch R. (2010). Metabolic Reprogramming, Caloric Restriction and Aging. Trends Endocrinol. Metab..

[45] Chou S.-H., Lee Y.-C., Huang C.-F., Wang Y.-R., Yu H.-P., Lau Y.-T. (2010). Gender-Specific Effects of Caloric Restriction on the Balance of Vascular Nitric Oxide and Superoxide Radical. Cardiovasc. Res..

[46] Ketonen J., Pilvi T., Mervaala E. (2010). Caloric Restriction Reverses High-Fat Diet-Induced Endothelial Dysfunction and Vascular Superoxide Production in C57Bl/6 Mice. Heart Vessels.

[47] Miyaki A., Maeda S., Yoshizawa M., Misono M., Saito Y., Sasai H., Endo T., Nakata Y., Tanaka K., Ajisaka R. (2009). Effect of Weight Reduction with Dietary Intervention on Arterial Distensibility and Endothelial Function in Obese Men. Angiology.

[48] Walford R.L., Harris S.B., Gunion M.W. (1992). The Calorically Restricted Low-Fat Nutrient-Dense Diet in Biosphere 2 Significantly Lowers Blood Glucose, Total Leukocyte Count, Cholesterol, and Blood Pressure in Humans. Proc. Natl. Acad. Sci. USA.

[49] Johnson M.L., Distelmaier K., Lanza I.R., Irving B.A., Robinson M.M., Konopka A.R., Shulman G.I., Nair K.S. (2016). Mechanism by Which Caloric Restriction Improves Insulin Sensitivity in Sedentary Obese Adults. Diabetes.

[50] Larson-Meyer D.E., Heilbronn L.K., Redman L.M., Newcomer B.R., Frisard M.I., Anton S., Smith S.R., Alfonso A., Ravussin E., the Pennington CALERIE Team (2006). Effect of Calorie Restriction With or Without Exercise on Insulin Sensitivity, β-Cell Function, Fat Cell Size, and Ectopic Lipid in Overweight Subjects. Diabetes Care.

[51] Fontana L., Villareal D.T., Weiss E.P., Racette S.B., Steger-May K., Klein S., Holloszy J.O. (2007). Calorie Restriction or Exercise: Effects on Coronary Heart Disease Risk Factors. A Randomized, Controlled Trial. Am. J. Physiol.-Endocrinol. Metab..

[52] Kostogrys R.B., Franczyk-Żarów M., Manterys A., Wybrańska I. (2018). Effect of Caloric Restriction on Liver Function in Young and Old ApoE/LDLr-/- Mice. Rocz. Panstw. Zakl. Hig..

[53] Villareal D.T. (2006). Bone Mineral Density Response to Caloric Restriction–Induced Weight Loss or Exercise-Induced Weight Loss: A Randomized Controlled Trial. Arch. Intern. Med..

[54] Wei S., Zhao J., Bai M., Li C., Zhang L., Chen Y. (2019). Comparison of Glycemic Improvement between Intermittent Calorie Restriction and Continuous Calorie Restriction in Diabetic Mice. Nutr. Metab..

[55] Kim C., Wellons M. (2023). Sex Hormones and Cardiovascular Disease in Relation to Menopause. Endocrinol. Metab. Clin. N. Am..

[56] Coylewright M., Reckelhoff J.F., Ouyang P. (2008). Menopause and Hypertension: An Age-Old Debate. Hypertension.

[57] Mercuro G., Zoncu S., Saiu F., Mascia M., Melis G.B., Rosano G.M.C. (2004). Menopause Induced by Oophorectomy Reveals a Role of Ovarian Estrogen on the Maintenance of Pressure Homeostasis. Maturitas.

[58] Murase T., Hattori T., Ohtake M., Nakashima C., Takatsu M., Murohara T., Nagata K. (2012). Effects of Estrogen on Cardiovascular Injury in Ovariectomized Female DahlS.Z- Lepr fa /Lepr fa Rats as a New Animal Model of Metabolic Syndrome. Hypertension.

[59] De Souza Nunes Faria M.S., Pimentel V.E., Helaehil J.V., Bertolo M.C., Santos N.T.H., Da Silva-Neto P.V., Thomazini B.F., De Oliveira C.A., Do Amaral M.E.C. (2022). Caloric Restriction Overcomes Pre-Diabetes and Hypertension Induced by a High Fat Diet and Renal Artery Stenosis. Mol. Biol. Rep..

[60] Çevikelli-Yakut Z.A., Özçelik R., Çevik Ö., Şener T.E., Şener G. (2021). Exercise and Caloric Restriction Improve Cardiovascular and Erectile Function in Rats with Metabolic Syndrome. Int. J. Impot. Res..

[61] Mager D.E., Wan R., Brown M., Cheng A., Wareski P., Abernethy D.R., Mattson M.P. (2006). Caloric Restriction and Intermittent Fasting Alter Spectral Measures of Heart Rate and Blood Pressure Variability in Rats. FASEB J..

[62] Schutte R., Thijs L., Asayama K., Boggia J., Li Y., Hansen T.W., Liu Y.-P., Kikuya M., Björklund-Bodegård K., Ohkubo T. (2013). Double Product Reflects the Predictive Power of Systolic Pressure in the General Population: Evidence from 9,937 Participants. Am. J. Hypertens..

[63] Timmers S., Konings E., Bilet L., Houtkooper R.H., van de Weijer T., Goossens G.H., Hoeks J., van der Krieken S., Ryu D., Kersten S. (2011). Calorie Restriction-like Effects of 30 Days of Resveratrol Supplementation on Energy Metabolism and Metabolic Profile in Obese Humans. Cell Metab..

[64] Abete P., Testa G., Ferrara N., De Santis D., Capaccio P., Viati L., Calabrese C., Cacciatore F., Longobardi G., Condorelli M. (2002). Cardioprotective Effect of Ischemic Preconditioning Is Preserved in Food-Restricted Senescent Rats. Am. J. Physiol.-Heart Circ. Physiol..

[65] Broderick T.L., Belke T., Driedzic W.R. (2002). Effects of chronic caloric restriction on mitochondrial respiration in the ischemic reperfused rat heart. Mol. Cell. Biochem..

[66] Guo Z., Wang M., Ying X., Yuan J., Wang C., Zhang W., Tian S., Yan X. (2023). Caloric Restriction Increases the Resistance of Aged Heart to Myocardial Ischemia/Reperfusion Injury via Modulating AMPK–SIRT1–PGC1a Energy Metabolism Pathway. Sci. Rep..

[67] Covell J.W., Ross J. (2011). Systolic and Diastolic Function (Mechanics) of the Intact Heart. Comprehensive Physiology.

[68] Hancock C.R., Han D., Higashida K., Kim S.H., Holloszy J.O. (2011). Does Calorie Restriction Induce Mitochondrial Biogenesis? A Reevaluation. FASEB J..

[69] Miller B.F., Robinson M.M., Bruss M.D., Hellerstein M., Hamilton K.L. (2012). A Comprehensive Assessment of Mitochondrial Protein Synthesis and Cellular Proliferation with Age and Caloric Restriction: Caloric Restriction and Mitochondrial Synthesis. Aging Cell.

[70] Hall J.E. (2015). Endocrinology of the Menopause. Endocrinol. Metab. Clin. N. Am..

[71] Martin S.J. (2011). Mitochondrial Fusion: Bax to the Fussure. Dev. Cell.

[72] Strehlow K., Rotter S., Wassmann S., Adam O., Grohé C., Laufs K., Böhm M., Nickenig G. (2003). Modulation of Antioxidant Enzyme Expression and Function by Estrogen. Circ. Res..

[73] Campos C., Casali K.R., Baraldi D., Conzatti A., Araújo A.S.D.R., Khaper N., Llesuy S., Rigatto K., Belló-Klein A. (2014). Efficacy of a Low Dose of Estrogen on Antioxidant Defenses and Heart Rate Variability. Oxid. Med. Cell. Longev..

[74] Felix A.C.S., Gastaldi A.C., Dutra S.G.V., de Freitas A., Philbois S.V., de Paula Facioli T., Silva V.D., Fares T.H., de Souza H. (2019). Early Ovarian Hormone Deprivation Increases Cardiac Contractility in Old Female Rats—Role of Physical Training. Auton. Neurosci. Basic Clin..

[75] Chen Y.-R., Zweier J.L. (2014). Cardiac Mitochondria and Reactive Oxygen Species Generation. Circ. Res..

[76] Ash C.E., Merry B.J. (2011). The Molecular Basis by Which Dietary Restricted Feeding Reduces Mitochondrial Reactive Oxygen Species Generation. Mech. Ageing Dev..

[77] Merry B.J. (2004). Oxidative Stress and Mitochondrial Function with Aging—The Effects of Calorie Restriction. Aging Cell.

[78] Yu B.P. (1996). Aging and Oxidative Stress: Modulation by Dietary Restriction. Free Radic. Biol. Med..

[79] Aydin C., Ince E., Koparan S., Cangul I.T., Naziroglu M., Ak F. (2007). Protective Effects of Long Term Dietary Restriction on Swimming Exercise-Induced Oxidative Stress in the Liver, Heart and Kidney of Rat. Cell Biochem. Funct..

[80] Aydin C., Sonat F., Sahin S.K., Cangul I.T., Ozkaya G. (2009). Long Term Dietary Restriction Ameliorates Swimming Exercise-Induced Oxidative Stress in Brain and Lung of Middle-Aged Rat. Indian J. Exp. Biol..

[81] Chen L.H., Saxon-Kelley D.M., Snyder D.L. (1996). Effects of Age and Dietary Restriction on Liver Endogenous Antioxidant Defenses in Male Lobund-Wistar Rats. Age.

[82] Bergamini E., Cavallini G., Donati A., Gori Z. (2007). The Role of Autophagy in Aging: Its Essential Part in the Anti-Aging Mechanism of Caloric Restriction. Ann. N. Y. Acad. Sci..

[83] Cavallini G., Donati A., Gori Z., Pollera M., Bergamini E. (2001). The Protection of Rat Liver Autophagic Proteolysis from the Age-Related Decline Co-Varies with the Duration of Anti-Ageing Food Restriction. Exp. Gerontol..

[84] Shinmura K., Tamaki K., Sano M., Murata M., Yamakawa H., Ishida H., Fukuda K. (2011). Impact of Long-Term Caloric Restriction on Cardiac Senescence: Caloric Restriction Ameliorates Cardiac Diastolic Dysfunction Associated with Aging. J. Mol. Cell. Cardiol..

[85] Tannous C., Booz G.W., Altara R., Muhieddine D.H., Mericskay M., Refaat M.M., Zouein F.A. (2021). Nicotinamide Adenine Dinucleotide: Biosynthesis, Consumption and Therapeutic Role in Cardiac Diseases. Acta Physiol..

[86] Wang Y.-H., Li S.-A., Huang C.-H., Su H.-H., Chen Y.-H., Chang J.T., Huang S.-S. (2018). Sirt1 Activation by Post-Ischemic Treatment With Lumbrokinase Protects Against Myocardial Ischemia-Reperfusion Injury. Front. Pharmacol..

[87] Wu Q.-J., Zhang T.-N., Chen H.-H., Yu X.-F., Lv J.-L., Liu Y.-Y., Liu Y.-S., Zheng G., Zhao J.-Q., Wei Y.-F. (2022). The Sirtuin Family in Health and Disease. Signal Transduct. Target. Ther..

[88] Yamamoto T., Tamaki K., Shirakawa K., Ito K., Yan X., Katsumata Y., Anzai A., Matsuhashi T., Endo J., Inaba T. (2016). Cardiac Sirt1 Mediates the Cardioprotective Effect of Caloric Restriction by Suppressing Local Complement System Activation after Ischemia-Reperfusion. Am. J. Physiol.-Heart Circ. Physiol..

[89] Zhang J., Zhang W., Gao X., Zhao Y., Chen D., Xu N., Pu H., Stetler R.A., Gao Y. (2019). Preconditioning with Partial Caloric Restriction Confers Long-Term Protection against Grey and White Matter Injury after Transient Focal Ischemia. J. Cereb. Blood Flow Metab..

[90] Cohen H.Y., Miller C., Bitterman K.J., Wall N.R., Hekking B., Kessler B., Howitz K.T., Gorospe M., De Cabo R., Sinclair D.A. (2004). Calorie Restriction Promotes Mammalian Cell Survival by Inducing the SIRT1 Deacetylase. Science.

[91] Mansur A.P., Roggerio A., Goes M.F.S., Avakian S.D., Leal D.P., Maranhão R.C., Strunz C.M.C. (2017). Serum Concentrations and Gene Expression of Sirtuin 1 in Healthy and Slightly Overweight Subjects after Caloric Restriction or Resveratrol Supplementation: A Randomized Trial. Int. J. Cardiol..

[92] Planavila A., Dominguez E., Navarro M., Vinciguerra M., Iglesias R., Giralt M., Lope-Piedrafita S., Ruberte J., Villarroya F. (2012). Dilated Cardiomyopathy and Mitochondrial Dysfunction in Sirt1-Deficient Mice: A Role for Sirt1-Mef2 in Adult Heart. J. Mol. Cell. Cardiol..

[93] Lin S.-J., Ford E., Haigis M., Liszt G., Guarente L. (2004). Calorie Restriction Extends Yeast Life Span by Lowering the Level of NADH. Genes Dev..

[94] Covarrubias A.J., Perrone R., Grozio A., Verdin E. (2021). NAD+ Metabolism and Its Roles in Cellular Processes during Ageing. Nat. Rev. Mol. Cell Biol..

[95] Matsushima S., Sadoshima J. (2015). The Role of Sirtuins in Cardiac Disease. Am. J. Physiol.-Heart Circ. Physiol..

[96] Watroba M., Szukiewicz D. (2021). Sirtuins at the Service of Healthy Longevity. Front. Physiol..

[97] Ong S., Hall A.R., Dongworth R.K., Kalkhoran S., Pyakurel A., Scorrano L., Hausenloy D.J. (2015). Akt Protects the Heart against Ischaemia-Reperfusion Injury by Modulating Mitochondrial Morphology. Thromb. Haemost..

