# Supplementary material for: Intense Caloric Restriction from Birth Protects the Heart Against Ischemia/Reperfusion Injury and Reduces Reactive Oxygen Species in Ovariectomized Rats
Source: Antioxidants (Basel). 2025 Jan 31;14(2):169. doi: 10.3390/antiox14020169 (PMC11851507; doi:10.3390/antiox14020169)
Supplement: Supplementary file 1 [file antioxidants-14-00169-s001.zip › antioxidants-3426709-supplementary.docx]

Table S1: Body weight

|  | Control Group (n=10) | | Restricted Group (n=10) | |  |  |
| --- | --- | --- | --- | --- | --- | --- |
| Weeks-old | Mean | SD | Mean | SD | Difference | *p* value |
| 0 | 6.836 | 0.5778 | 6.500 | 1.039 | 0.3360 | 0.383256 |
| 1 | 16.23 | 1.572 | 13.57 | 1.685 | 2.660 | 0.001831 |
| 2 | 30.48 | 3.018 | 20.43 | 3.09 | 10.05 | <0.000001 |
| 3 | 53.39 | 12.55 | 31.85 | 7.083 | 21.54 | 0.000168 |
| 4 | 91.79 | 15.27 | 54.19 | 10.45 | 37.60 | 0.000005 |
| 5 | 133.1 | 8.268 | 79.14 | 10.01 | 53.96 | <0.000001 |
| 6 | 156.9 | 13.44 | 100.9 | 11.92 | 56.00 | <0.000001 |
| 7 | 182.1 | 14.42 | 110.5 | 10.39 | 71.60 | <0.000001 |
| 8 | 197.2 | 22.41 | 122.0 | 10.33 | 75.20 | <0.000001 |
| 9 | 215.0 | 14.72 | 134.0 | 11.25 | 81.00 | <0.000001 |
| 10 | 225.5 | 11.41 | 144.0 | 10.22 | 81.50 | <0.000001 |
| 11 | 230.0 | 14.14 | 151.5 | 10.81 | 78.50 | <0.000001 |
| 12 | 239.5 | 12.79 | 153.0 | 15.49 | 86.50 | <0.000001 |
